# Basal cell carcinoma treated successfully with combined CO2 laser and photodynamic therapy in a renal transplant patient: a case report

**DOI:** 10.4076/1757-1626-2-7920

**Published:** 2009-08-11

**Authors:** Kayvan Shokrollahi, Nicholas J Marsden, Iain S Whitaker, William James, Maxwell SC Murison

**Affiliations:** Department of Burns and Plastic Surgery, Morriston HospitalHeol Maes Eglwys, Morriston, Swansea, SA6 6NLUK

## Abstract

Renal transplant recipients are at significantly greater risk of developing skin malignancies due to combination immunosuppressive therapy. A significant number of patients present with lesions needing excision at multiple outpatient follow-up visits. For basal cell carcinoma, we have recently described how combining CO_2_ laser with Photodynamic therapy greatly increases the efficacy of long-term tumour clearance compared with each modality alone. We present a case of a 66-year-old renal transplant therapy patient who repeatedly presents with new skin malignancies, in whom we treated successfully with Laser-Photodynamic therapy in a see-and-treat setting. This therapy offers patients the possibility of better cosmetic and functional results whilst obviating the need for repeated surgery. Other pre-cancerous lesions such as solar keratoses are prevalent in this patient group and respond extremely well to Photodynamic therapy monotherapy. We propose a regular clinic for renal transplant patients in a laser facility equipped with CO_2_ laser and Photodynamic therapy, histopathology and punch-biopsy materials. This strategy allows simple and effective treatment of multiple lesions simultaneously, avoidance of numerous operations, avoidance of non-essential outpatient appointments that result in booking furthers visits for treatment, whilst facilitating diagnostic biopsies of potentially malignant lesions. We outline a care pathway for a see-and-treat clinic that implements this novel treatment modality improving the care of this unique patient population.

## Introduction

Basal cell carcinoma (BCC) is the most common cancer in many parts of the world, and is increasing in incidence in the UK [1]. Renal transplant recipients are at significantly greater risk of developing skin malignancies due to combination immunosuppressive therapy [2]. Surveillance in these renal transplant patients for BCC and other skin malignancies is important [3]. The morbidity associated with BCCs is related to local tissue invasion and destruction as well as aesthetic deformity due to both the disease itself and the consequences of treatment. Treatment usually necessitates excision of these lesions with an appropriate margin guided by published recommendations [4], often resulting in less than ideal cosmetic outcomes especially if local flaps or skin grafts are required for reconstruction. Furthermore, in certain anatomical areas such as around the eyelids, direct closure after excision of these lesions is often precluded due to the potential distortion of important landmarks leading to complications such as ectropion or epiphora. Many other treatment options exist for BCC. Topical pharmacological therapy, cryotherapy, radiotherapy, PDT and laser have all been used [5,6]. Non-surgical treatments remain common and many are managed in a dermatology setting.

Photodynamic therapy (PDT) and carbon dioxide laser, when used as monotherapy, have been successfully used to treat both malignant and non-malignant cutaneous lesions, including basal cell carcinoma [7] with the greatest success in the superficial histological subtype of basal cell carcinoma, and those of the nodular variety that are small in size. Other pre-cancerous lesions such as solar keratoses are prevalent in this patient group and respond extremely well to Photodynamic therapy monotherapy [8]. These modalities when used alone have a number of limitations when compared to surgical excision, including a limited depth of penetration of PDT (2 mm absorption), which potentially limits the efficacy of treatment for nodular BCCs greater than this thickness, or which are deeply invasive. We have shown great success through combining these modalities by the initial use of the CO2 laser to debulk lesions so as to enable subsequent PDT to be effective on any residual tumour [7]. The use of this new strategy opens up new possibilities in the treatment of basal cell carcinomata both clinically and logistically. To date, we have treated almost one hundred patients without recurrence, with follow-up times of up to three years. Many of our patients who are renal transplant recipients develop skin tumours requiring treatment at almost every follow-up visit.

## Case presentation

A 66-year-old British Caucasian man was referred to our laser clinic for treatment of multiple skin malignancies, as a result of his renal transplant therapy. He was originally diagnosed with polycystic kidney disease at the age of 36, and went on to undergo regular renal dialysis, until a kidney transplant was carried out in March 1999. His other co-morbidities include angina, hypertension and hypercholesterolaemia. He has been taking cyclosporine 75 mg BD for the past 10 years following his transplant. He has had multiple skin lesions develop over this time, treated by a multidisciplinary team including his GP, dermatologist and more recently by the plastic surgeons. Around 40 lesions have been treated with cryotherapy, and 12 lesions surgically excised. He was referred to our laser clinic with 5 separate skin lesions on his back, clinically diagnosed as BCC. After careful discussion and consent from the patient, we decided upon combined laser and PDT for treatment of this gentleman’s skin malignancies.

### Combined laser and PDT method

**Figure 1.**
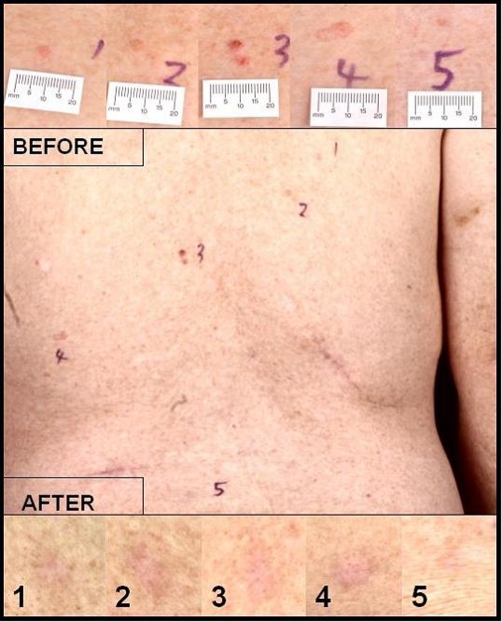
Laser-PDT treatment of 5 simultaneous lesions on a renal transplant recipient’s back, showing results 3 years post treatment, with no sign of recurrence.

We use an Ultra Pulse^®^ CO_2_ Laser set at 150 mJ, 10 Hz, with a 2 mm collimated beam to vaporise BCCs of the nodular variety, whilst maintaining a bloodless field with no eschar. PDT is then carried out using the photosensitizing agent Methyl Aminolevulinate (METVIX®) and illuminated with the Aktilite 16 LED lamp at 631 nm, 37 J/cm^2^ for 7 minutes 24 seconds. In our unit we have treated 90 patients over a 3-year period with no recurrences using this technique. Figure 1 illustrates our patient, who we treated successfully with laser-PDT in a see-and-treat setting. Although there is no evidence of recurrence of previously treated lesions after 3 years follow-up, he continues to present with new lesions elsewhere.

Our strategy for the management of this group of patients is as follows: Basal cell carcinomas are treated with combination laser-PDT, and biopsies undertaken simultaneously when necessary. Actinic keratoses (solar keratoses - SKs) are treated with PDT alone, unless they are very keratotic (shaved or CO_2_ lasered) or suspicion exists that they are well differentiated SCCs at which time a biopsy is undertaken. Biopsy confirmed SCCs are treated with wide local excision, as are recurrent lesions that have previously been treated by any modality.

The incidence of squamous cell carcinoma, in immunocompromised renal transplant recipients is considerably higher compared with the normal population (up to 100-fold), and in contrast to the normal population, are more prominent than BCCs [9]. SCC growth in transplant patients is usually more rapid and aggressive and if there is marked local tissue invasion and regional lymph node metastasis, can prove life threatening. Treatment is often difficult and recurrence common, therefore laser-PDT therapy is not recommended in these patients, and all SCC should be treated with wide local surgical excision.

## Discussion

One may argue that surgical excision of basal cell carcinomata is undertaken too frequently with sub-optimal cosmetic and functional results. An elliptical excision of a well circumscribed basal cell carcinoma with the recommended 4 mm margin results in a substantial defect which, even when closed directly without resorting to a skin graft or local flap reconstruction, leads to a significant scar much larger than the original lesion. Furthermore, the benefit of histological confirmation of diagnosis or clearance should not be given undue weight: histological confirmation of tumour clearance is unavailable for many widely accepted methods of treatment, particularly prevalent in the dermatology setting, and basal cell carcinomata almost never metastasize [10] or threaten life. One must therefore ask the question as to whether this additional information warrants a cosmetically suboptimal surgical treatment, especially in cosmetically sensitive areas such as on the face. This is particularly pertinent as studies have shown that poor cosmesis has the most significant negative impact on the quality of life of these patients [11]. The disadvantages of surgical excision are compounded for renal transplant patients in whom these lesions are numerous and frequently occurring, making a routine surgical strategy even less ideal.

Many problematic lesions in renal transplant patients are solar keratoses or Bowen’s disease. PDT with or without laser is already recognised as one of the best modalities for treatment of these lesions [12], especially in this patient group [13]. Tantalisingly, evidence is emerging that treating at-risk areas of skin with PDT can prevent the emergence of non-melanoma skin cancers [10]. This suggests that a Laser-PDT strategy may also reduce the incidence of other lesions in adjacent areas where treatment has been undertaken.

## Conclusion

A regular see-and-treat Laser-PDT equipped clinic provides holistic care for patients with numerous non-melanoma skin cancers providing a surveillance and treatment service and providing treatments with better cosmetic outcomes and with low recurrence rates.

## Abbreviations

BCC: Basal Cell Carcinoma
GP: General Practitioner
LED: Light Emitting Diode
PDT: Photodynamic Therapy
SCC: Squamous Cell Carcinoma
SK: Solar Keratoses
